# Dosimetric study on the effect of minimum distance between optimization structures and planning target volume on small intestine dose in cervical cancer VMAT plans

**DOI:** 10.1097/MD.0000000000044715

**Published:** 2025-09-26

**Authors:** Rui Ban, Jing Zeng, Jinlong Hao

**Affiliations:** a Department of Gynecology, Department of Gynecological Oncology, Tianjin Institute of Gynaecology Obsterics, Tianjin Central Hospital of Gynaecology Obsterics, Tianjin, China.

**Keywords:** cervical cancer, dose to small intestine, optimization structure, volumetric modulated arc therapy (VMAT)

## Abstract

Cervical cancer radiotherapy often faces challenges in managing gastrointestinal toxicities, particularly due to the dose received by the small intestine. This study investigates the impact of optimization structure margins (1.2–2.4 cm) on reducing small intestine dose and improving plan quality in patients with mean small intestine doses (D_mean_INT_) exceeding 25 Gy. A total of 27 cervical cancer patients treated with volumetric modulated arc therapy were retrospectively analyzed. Iterative constraint adjustments were applied to optimization structures at varying distances from the planning target volume (PTV). Five optimization margins (0.6, 1.2, 1.8, 2.4, and 3.0 cm) were created by volumetrically excluding small intestine regions proximal to PTV, with iterative dose constraints. Each plan was named RrIi, where r represents the cutting distance of the optimization structure and i represents the iteration number (ranging from 4–8). Results demonstrated that all optimized plans achieved significant reductions in D_mean_INT_ compared to the original plans (ORI group), with reductions ranging from 1.21 to 1.83 Gy (*P* < .001). The R_1.2I_5.5 protocol achieved the most favorable balance, reducing D_mean_INT_ by 1.23 Gy, corresponding to a 15% to 22% relative risk reduction for Grade ≥ 2 enteritis. Smaller margins (1.2 cm) effectively reduced intermediate-high-dose regions (e.g., V_40Gy_), while larger margins (2.4 cm) better controlled low-dose regions (e.g., V_15Gy_), supporting tailored clinical decision-making based on patient characteristics. In conclusion, optimization structure margins of 1.2 to 2.4 cm provide a clinically meaningful framework for reducing small intestine dose while preserving PTV coverage, advancing cervical cancer radiotherapy planning.

## 1. Introduction

Cervical cancer is the most common malignant tumor of the female reproductive system. Radiotherapy is one of the fundamental treatment methods for cervical cancer.^[1]^ However, radiotherapy is often accompanied by gastrointestinal toxicities, with the dose received by the small intestine being a significant contributing factor.^[2–6]^ In 2017, the American Radiation Therapy Oncology Group 1203 study reported that 51.9% of women undergoing conventional radiotherapy and 33.7% of those receiving intensity-modulated radiotherapy (IMRT) experienced frequent or persistent diarrhea during the acute phase. Severe cases often led to treatment interruptions, adversely affecting the efficacy of radiotherapy.^[7]^ According to the 2010 international guidelines for organ dose limits in radiotherapy (Quantitative Analysis of Normal Tissue Effects in the Clinic), the incidence of ≥ Grade 3 acute intestinal adverse reactions should be kept below 10%.^[8]^

This study explores the impact of the minimum distance between optimization structures and the planning target volume (PTV) on reducing the radiation dose to the small intestine and improving overall plan quality when optimization structures are employed.

Currently, volumetric modulated arc therapy (VMAT) is widely used for cervical cancer radiotherapy.^[9–11]^ In VMAT planning optimization, the use of additional optimization structures and the application of dose constraints are common strategies to reduce doses to organs at risk. We hypothesize that optimization margins between 1.2 and 2.4 cm could achieve an optimal balance between sparing the small intestine and maintaining adequate target coverage.

## 2. Participants and methods

### 2.1. Patient characteristics

This study selected 27 patients with cervical cancer who underwent radical radiotherapy at Tianjin Central Obstetrics and Gynecology Hospital from 01/01/2022 to 31/01/2024. Data were accessed for research purposes on 01/06/2024. As shown in Table 1, the patients’ ages ranged from 34 to 75 years, with a mean age of 57 years. According to the International Federation of Gynecology and Obstetrics (FIGO) staging criteria, the clinical stages of the patients were between IIB and IIIB, with pathological diagnoses of moderately to poorly differentiated squamous cell carcinoma. The cases selected for this study had relatively high D_mean_INT_ during clinical treatment, with D_mean_INT_ values ranging from 26.8 Gy to 28.4 Gy, averaging 27.68 Gy. This study strictly adhered to ethical standards in medicine. As a retrospective study, it was granted an exemption from obtaining informed consent from patients in the ethical review process, receiving approval from the hospital’s ethics committee (Ethics Review No. 2023KY045).

**Table 1:** Patient characteristics.

Characteristic	Value
Age mean (range)	57 (34–75)
Histology SCC, n	27
Histologic grade G2–3, n	27
FIGO 2018 stage, n	
IIB	20
IIIB	7
Dmean_INT in Gy (range)	27.68 (26.8–28.4)

Dmean_INT = mean dose to the small intestine, FIGO = International Federation of Gynecology and Obstetrics, SCC = squamous cell carcinoma.

### 2.2. Bowel and bladder preparation protocols

Patients are usually required to keep their bladder full and their rectum empty before treatment. Before each treatment appointment, patients need to drink enough water about 30 minutes prior to the appointment to ensure bladder filling, which typically includes drinking 400 mL of water. At the same time, patients should ensure they have a bowel movement beforehand to keep the rectum empty, thereby reducing interference during the treatment. Additionally, dietary preparation is also important. Patients are advised to follow a low-residue diet before treatment to reduce the amount of feces and gas in the intestines. Adequate fluid intake and regular eating habits help maintain normal bowel function. If necessary, doctors may recommend the use of mild laxatives, such as Dulcolax, in the few days before CT simulation or radiotherapy to help clear the rectum.

### 2.3. CT imaging and target volume delineation

Patients were positioned supine and immobilized using thermoplastic masks. CT imaging was performed with a Philips Brilliance CT Big Bore scanner, acquiring images with a slice thickness and gap of 5 mm. These images were then transferred to the treatment planning system for target volume delineation.

The clinician outlined the Clinical Target Volume (CTV), Gross Tumor Volume (GTV), and various organs at risk. The upper boundary of the CTV was located at the bifurcation of the abdominal aorta, while the lower boundary was defined by the inferior margin of the obturator foramen. The GTV was delineated following the guidelines,^[12]^ encompassing MRI-visible tumor extensions with 5 mm isotropic expansion accounting for microscopic spread. The CTV and GTV were expanded 0.7 cm in the craniocaudal direction and 0.5 cm in other directions to obtain the PTV and Planning GTV. Organs at risk included the bladder, rectum, small intestine, pelvis bone, femoral head, and spinal cord. The small intestine was delineated using the peritoneal cavity outlining method.^[13,14]^

### 2.4. Treatment planning

**Figure 1.**
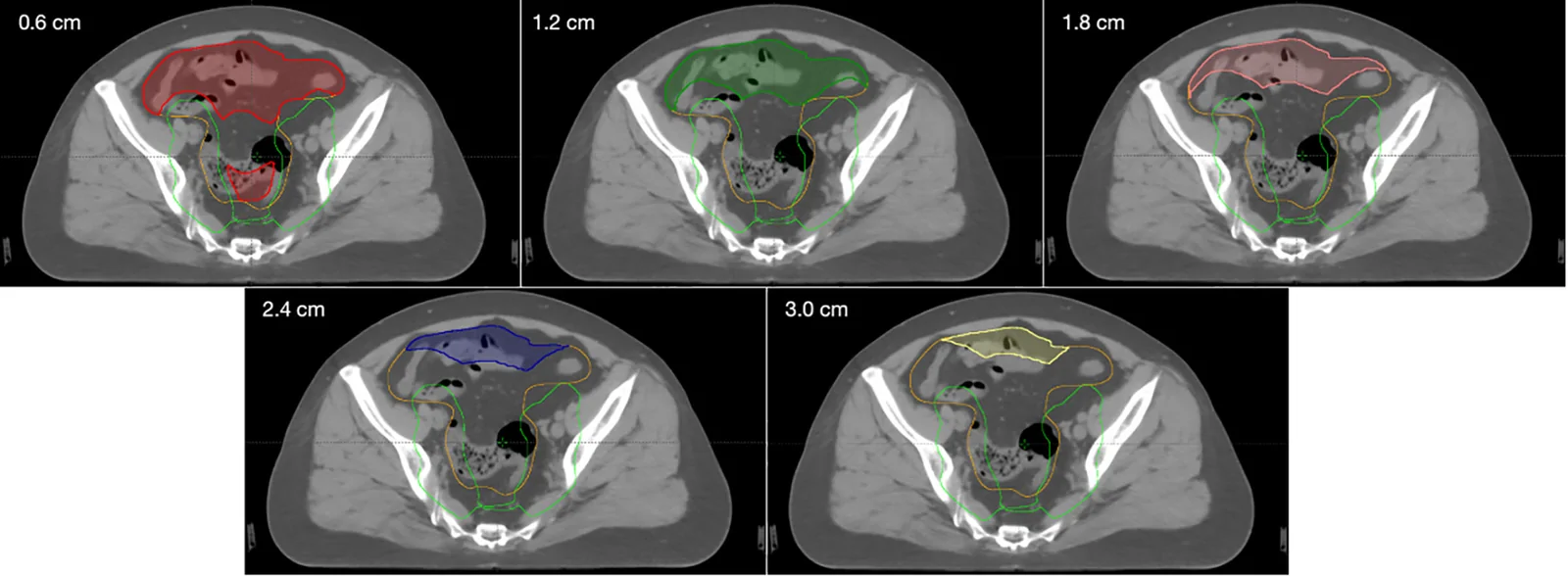
Schematic diagram of 5 optimization structures. Eclipse treatment planning system, v 13.6, https://www.varian.com/zh-hans/products/radiosurgery/treatment-planning.

Treatment planning was conducted using the Varian Eclipse 13.6 planning system. The prescribed dose for the PTV was 48.6 Gy (27 fractions of 1.8 Gy), with the planning GTV receiving a simultaneous boost to 50 Gy. A single medical physicist optimized the plan. During optimization, strict dose constraints were applied to the small intestine’s dose-volume histogram (DVH) to minimize radiation exposure. The resulting plans, which were used clinically during patient treatment, are referred to as the ORI group. Subsequently, optimization structures were created by cutting the small intestine from the PTV outline at specified distances, which represented the minimum distance between the optimization structures and the PTV (denoted as r). As shown in Figure 1, 5 optimization structures were generated corresponding to cutting distances of 0.6, 1.2, 1.8, 2.4, and 3.0 cm. The green structure represents the PTV, the orange structure represents the small intestine, and the remaining colors represent the optimization structures. The cutting distances for the optimization structures are indicated as follows: red for 0.6 cm, dark green for 1.2 cm, pink for 1.8 cm, dark blue for 2.4 cm, and yellow for 3.0 cm. Each plan only applied dose constraints to 1 specific r optimizationstructure. An iterative rule was designed to progressively enhance the constraint strength on the optimizationstructures. This paper designs a set of iterative rules to gradually enhance the constraint strength on auxiliary structures. The first iteration set the the doses achieved by 50% and 20% of the volume of the optimization structures (D_50%_ and D_20%_) in the ORI plan as optimization targets for D_40%_ and D_10%_, respectively, with weights set to 100. Starting from the second iteration, 2 operations were alternated: reducing the dose targets for D_40%_ and D_10%_ by 1.5 Gy based on the previous iteration’s values; and increasing the weights for D_40%_ and D_10%_ by 50 based on the previous iteration’s values. Iterations ceased when either: The mean doses (D_mean_) reduction plateaued (<0.3 Gy change over 2 iterations), or The 95% volume (V95%) of the PTV did not meet the institutional constraint of 98%. This alternating approach gradually enhanced the dose constraints on the optimizationstructures. Specific values for the dose targets and weights at different iteration steps are shown in Table 2. It is noteworthy that the constraint strength for the PTV’s dose limits V_98%_ and V_95%_ was set to 300. Each iteration only modified the optimization parameters for the optimizationstructures without inheriting the final beam state from the previous optimization step. Thus, after setting the constraints for the optimizationstructures, the entire plan optimization was restarted. Multiple VMAT plans were generated through iterations, naming each plan according to the cutting distance and iteration number as RrIi, where r represents the cutting distance (0.6, 1.2, 1.8, 2.4, or 3.0 cm) and i represents the iteration number (ranging from 1 to 8). An additional adjustment was made to the constraint strength for the *R* = 1.2 cm optimizationstructure, resulting in the R_1.2I_5.5 group, which had a constraint strength between R_1.2I_5 and R_1.2I_6. Other settings for each plan (such as collimator angles, tungsten leaf width and position, optimization targets and weights for other structures, etc) remained the same as in the ORI group.

**Table 2:** Dose constraints and weights for optimizationstructures at each iteration step.

Iteration step	Optimization goal of *D*_40%_ (Gy)	Optimization priority of *D*_40%_	Optimization goal of *D*_10%_ (Gy)	Optimization priority of *D*_10%_
1	*D*_50%_ORI_	100	*D*_20%_ORI_	100
2	*D*_50%_ORI_ – 1.5	100	*D*_20%_ORI_ – 1.5	100
3	*D*_50%_ORI_ – 1.5	150	*D*_20%_ORI_ – 1.5	150
4	*D*_50%_ORI_ – 3.0	150	*D*_20%_ORI_ – 3.0	150
5	*D*_50%_ORI_ – 3.0	200	*D*_20%_ORI_ – 3.0	200
5.5	*D*_50%_ORI_ – 3.75	200	*D*_20%_ORI_ – 3.75	200
6	*D*_50%_ORI_ – 4.5	200	*D*_20%_ORI_ – 4.5	200
7	*D*_50%_ORI_ – 4.5	250	*D*_20%_ORI_ – 4.5	250
8	*D*_50%_ORI_ – 6.0	250	*D*_20%_ORI_ – 6.0	250

D50%_ORI and D20%_ORI refer to the doses achieved by 50% and 20% of the volume of the optimization structures, respectively, in the ORI group plan.

### 2.5. Dosimetric evaluation

All plans were normalized to ensure that 95% of the volume receiving the prescribed dose of 48.6 Gy (V48.6Gy) was consistent across all groups for comparison. Due to the high overlap between the small intestine and PTV in the selected cases, clinical practice allows for some degree of underdosing of the PTV to ensure that the small intestine dose does not exceed the limits. Therefore, the normalization of the plans in this study was set to 85% to 95% rather than uniformly to 95%, while still ensuring that the V_48.6Gy_ values for each case were consistent across groups. The evaluation indices for the target volume included Normalization Rate (NR), Homogeneity Index (HI), and Conformity Index (CI). Normalization usually refers to standardizing the dose distribution to a reference point to compare different treatment plans and schemes. This normalization allows clinicians to better assess the effectiveness of different plans and make reasonable dose allocations. The NR is defined as the ratio of doses between pre-normalization and post-normalization isocentric points. The HI is defined as HI = (D_2%_–D_98%_)/ D_50%_, where D_2%_, D_50%_, and D_98%_ represent the doses received by 2%, 50%, and 98% of the PTV volume, respectively. The CI is defined as CI = (V_Tref_/ V_T_) × (V_Tref_/ V_ref_), where V_Tref_ is the volume of the PTV encompassed by the 95% isodose line, V_T_ is the target volume, and V_ref_ is the volume encompassed by the 95% isodose line. Values for HI and CI range from 0 to 1, with values closer to 0 indicating better dose uniformity and values closer to 1 indicating better dose conformity.

Regarding organs at risk, this study focused on the dose to the small intestine, comparing the average dose D_mean_INT_ and DVH parameters V_15Gy_, V_30Gy_, V_40Gy_, V_45Gy_, where V_nGy_ represents the percentage of the total volume receiving n Gy. Additionally, the average doses D_mean_BLA_ and D_mean_REC_ for the bladder and rectum were compared.

### 2.6. Statistical analysis

Data analysis was performed using IBM SPSS Statistics 21 software. As this study involved comparisons between multiple groups, one-way ANOVA was used to determine whether significant differences existed among the groups. For subsequent pairwise comparisons, Dunnett’s t-test was used to compare D_mean_INT_ against the ORI group, while the SNK-q test was employed for comparisons among multiple groups. The significance level was set at α = 0.05. In Dunnett’s t-test, *P* < .05 indicates statistical significance. In the SNK-q test, differences between any 2 plans were assessed based on their subset classifications; if 2 plans belonged to the same subset, the difference was not statistically significant; otherwise, it was considered significant.

## 3. Results

### 3.1. Comparison of mean dose to the small intestine and normalization rate across plans

**Figure 2.**
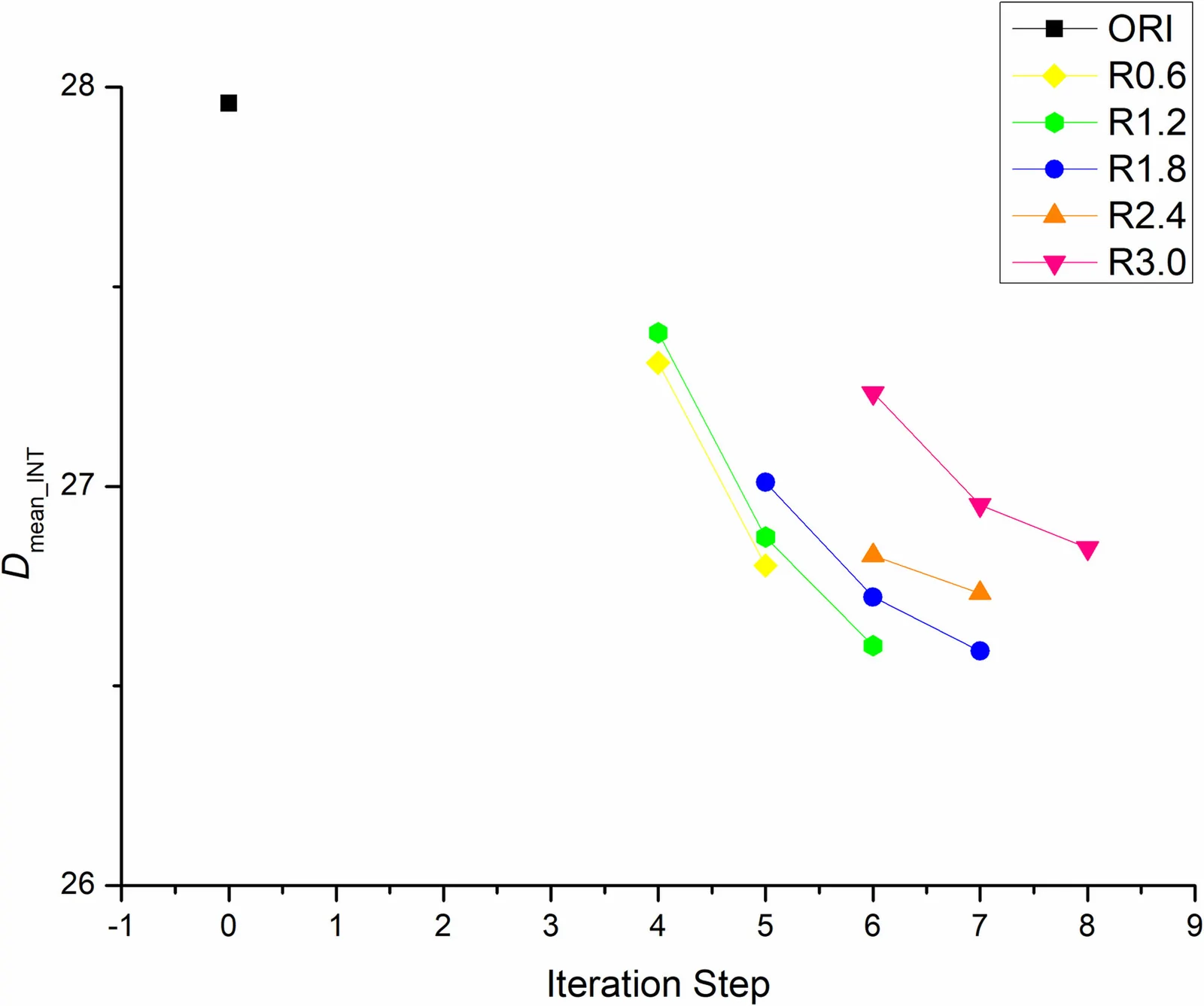
Mean dose to the small intestine for each group by cutting distance and iteration number.

Table 3 presents the specific values of D_mean_INT_ for the plans with dose constraints applied to the optimizationstructures, along with comparisons to the original ORI plan. It is evident that after adding optimizationstructures and applying dose constraints, the D_mean_INT_ values for all groups were lower than that of the ORI group, with statistically significant differences (*P* < .05). Figure 2 illustrates the trend of D_mean_INT_ as a function of iteration number for fixed cutting distances. As shown, for each cutting distance, D_mean_INT_ consistently decreased with increasing iteration numbers (indicating progressively stronger constraints on the optimizationstructures). After fine-tuning the constraint strength, we selected several plans with similar D_mean_INT_ values across different cutting distances for further comparison of NRs. Table 4 provides the comparative results for the plans R_0.6I_5, R_1.2I_5.5, R_1.8I_6, R_2.4I_7, R_3.0I_7, and R_3.0I_8. It can be seen that the plans R_0.6I_5, R_1.2I_5.5, R_1.8I_6, and R_2.4I_7 were part of the same subset in both D_mean_INT_ and NR comparisons, indicating no statistically significant differences among them. In the case of *R* = 3.0 cm, the R_3.0I_7 plan had a NR that was part of the same subset as R_0.6I_5, R_1.2I_5.5, R_1.8I_6, and R_2.4I_7, indicating no significant difference; however, in terms of D_mean_INT_, it was not part of the same subset, suggesting that R_3.0I_7 had a significantly higher D_mean_INT_ compared to the latter 4 plans, with a statistically significant difference. The R_3.0I_8 plan, on the other hand, was part of the same subset as R_0.6I_5, R_1.2I_5.5, R_1.8I_6, and R_2.4I_7 in terms of D_mean_INT_, indicating no significant difference, but it was not part of the same subset as R_1.2I_5.5 and R_1.8I_6 in terms of NR, indicating that its NR was significantly lower than these 2 groups, with a statistically significant difference. In summary, by adjusting the constraint strength, the plans R_0.6I_5, R_1.2I_5.5, R_1.8I_6, and R_2.4I_7 maintained consistency in both D_mean_INT_ and NR, with no significant differences among them. However, when *R* = 3.0 cm, achieving a D_mean_INT_ comparable to the other 4 groups resulted in a relatively low NR; conversely, ensuring a NR comparable to the others resulted in a relatively high D_mean_INT_. Thus, it is suggested that cutting distances of 0.6, 1.2, 1.8, or 2.4 cm are preferable over 3.0 cm for reducing small intestine radiation exposure. All optimized plans achieved statistically significant D_mean_INT_ reductions versus ORI (*P* < .001, Cohen d = 1.47–1.62), with R_1.2I_5.5 showing the largest effect size (d = 1.52, 95% CI 1.21–1.83).

**Table 3:** Mean dose to small intestine across optimization strategies with statistical comparisons.

	Dmean_INT (Gy)	Δ vs ORI (95% CI)	*P*-value	Cohen d (95% CI)
ORI	27.96 ± 0.56	–	–	–
R_0.6I_4	27.31 ± 0.75	−0.65 (−0.88 to −0.42)	<0.001	0.97 (0.62–1.32)
R_0.6I_5	26.80 ± 0.78	−1.16 (−1.42 to −0.90)	<0.001	1.47 (1.10–1.84)
R_1.2I_4	27.39 ± 0.76	−0.57 (−0.81 to −0.33)	<0.001	0.81 (0.47–1.15)
R_1.2I_5	26.87 ± 0.76	−1.09 (−1.34 to −0.84)	<0.001	1.41 (1.04–1.78)
R_1.2I_5.5	26.73 ± 0.80	−1.23 (−1.50 to −0.96)	<0.001	1.52 (1.21–1.83)
R_1.2I_6	26.60 ± 0.82	−1.36 (−1.64 to −1.08)	<0.001	1.64 (1.31–1.97)
R_1.8I_5	27.01 ± 0.67	−0.95 (−1.17 to −0.73)	<0.001	1.33 (1.00–1.66)
R_1.8I_6	26.72 ± 0.76	−1.24 (−1.49 to −0.99)	<0.001	1.53 (1.18–1.88)
R_1.8I_7	26.59 ± 0.76	−1.37 (−1.62 to −1.12)	<0.001	1.68 (1.33–2.03)
R_2.4I_6	26.83 ± 0.74	−1.13 (−1.37 to −0.89)	<0.001	1.44 (1.08–1.80)
R_2.4I_7	26.73 ± 0.78	−1.23 (−1.49 to −0.97)	<0.001	1.54 (1.19–1.89)
R_3.0I_6	27.15 ± 0.73	−0.81 (−1.04 to −0.58)	<0.001	1.15 (0.82–1.48)
R_3.0I_7	26.95 ± 0.74	−1.01 (−1.25 to −0.77)	<0.001	1.31 (0.97–1.65)
R_3.0I_8	26.84 ± 0.80	−1.12 (−1.39 to −0.85)	<0.001	1.43 (1.08–1.78)

1. Δ values represent differential dosimetric parameters between experimental plans (with optimizationstructure optimization) and original plans (ORI). Positive Δ indicates parameter increase, negative values denote reduction. 2. All p-values adjusted via Benjamini-Hochberg procedure (FDR < 5%). 3. Effect size interpretation (Cohen d): 0.2: Small; 0.5: Medium; ≥0.8: Large. 4. Data format: Mean ± SD (n = 27).

**Table 4:** Comparison of mean dose to the small intestine and normalization rate among the 7 plans.

	Dmean_INT (Gy)	NR (%)
ORI	27.96 ± 0.56	99.79 ± 0.18
R_0.6I_5	26.80 ± 0.78	99.41 ± 0.37
R_1.2I_5.5	26.73 ± 0.80	99.52 ± 0.32
R_1.8I_6	26.72 ± 0.76	99.56 ± 0.34
R_2.4I_7	26.73 ± 0.78	99.44 ± 0.46
R_3.0I_7	26.95 ± 0.74	99.58 ± 0.33
R_3.0I_8	26.84 ± 0.80	99.33 ± 0.41
Subsets	{R_1.8I_6, R_2.4I_7, R_1.2I_5.5}, {R_0.6I_5, R_3.0I_8};	{R_3.0I_8, R_0.6I_5, R_2.4I_7};
for α = 0.05	{R_3.0I_8, R_3.0I_7};	{R_0.6I_5, R_2.4I_7, R_1.2I_5.5, R_1.8I_6, R_3.0I_7};
	{ORI}	{ORI}

If 2 groups of plans belong to the same subset, it indicates that there is no statistically significant difference at the significance level of α = 0.05.

### 3.2. Comparison of other dosimetric parameters for PTV, bladder, and rectum

Table 5 shows the other dosimetric parameters for the plans R_0.6I_5, R_1.2I_5.5, R_1.8I_6, and R_2.4I_7, along with their comparative results. It can be observed that all 4 plans belong to the same subset in terms of CI, D_mean_BLA_ for the bladder, and D_mean_REC_ for the rectum, indicating no statistically significant differences among any pairwise comparisons. In terms of HI, the plans R_1.2I_5.5, R_1.8I_6, and R_2.4I_7 were part of the same subset, showing no significant differences among them. However, the R_0.6I_5 plan was not part of the same subset as the other 3 plans, indicating statistically significant differences, with R_0.6I_5 demonstrating significantly poorer dose uniformity in the target volume compared to the other 3 plans.

**Table 5:** Comparison of dosimetric parameters for PTV, bladder, and rectum among the 4 plans.

	R_0.6I_5	R_1.2I_5.5	R_1.8I_6	R_2.4I_7
*HI*	0.172 ± 0.038	0.169 ± 0.038	0.166 ± 0.038	0.167 ± 0.038
Subsets	{R_1.8I_6, R_2.4I_7, R_1.2I_5.5};			
for α = 0.05	{R_0.6I_5}			
*CI*	0.805 ± 0.028	0.802 ± 0.027	0.802 ± 0.026	0.801 ± 0.033
Subsets	{R_1.8I_6, R_2.4I_7, R_1.2I_5.5, R_0.6I_5}			
for α = 0.05				
*D*_mean_BLA_ (Gy)	41.79 ± 1.35	41.72 ± 1.33	41.71 ± 1.41	41.77 ± 1.35
Subsets	{R_1.8I_6, R_2.4I_7, R_1.2I_5.5, R_0.6I_5}			
for α = 0.05				
*D*_mean_REC_ (Gy)	39.45 ± 3.06	39.41 ± 3.10	39.33 ± 3.08	39.38 ± 3.12
Subsets	{R_1.8I_6, R_2.4I_7, R_1.2I_5.5, R_0.6I_5}			
for α = 0.05				

If 2 groups of plans belong to the same subset, it indicates that there is no statistically significant difference at the significance level of α = 0.05.

### 3.3. Comparison of small intestine DVH parameters across plans

Table 6 provides a comparison of small intestine dose volumes among the plans R_1.2I_5.5, R_1.8I_6, and R_2.4I_7. No statistically significant differences were found in V_45Gy_ among the 3 plans. In terms of V_15Gy_, R_1.8I_6 and R_2.4I_7 showed no significant differences and were significantly higher than R_1.2I_5.5. For V_30Gy_ and V_40Gy_, all pairwise comparisons among the 3 plans showed statistically significant differences. Overall, smaller cutting distances resulted in larger V_15Gy_ volumes, while larger cutting distances resulted in larger V_30Gy_ and V_40Gy_ volumes.

**Table 6:** Comparison of small intestine DVH parameters among the 3 plans.

	R_1.2I_5.5	R_1.8I_6	R_2.4I_7
*V*_15Gy_ (%)	69.68 ± 7.41	67.66 ± 5.30	66.34 ± 3.91
Subsets	{R_2.4I_7, R_1.8I_6};		
for α = 0.05	R_1.2I_5.5		
*V*_30Gy_ (%)	39.61 ± 4.03	41.22 ± 3.75	42.27 ± 3.47
Subsets	R_2.4I_7;		
for α = 0.05	R_1.8I_6;		
	R_1.2I_5.5		
*V*_40Gy_ (%)	28.40 ± 4.68	29.05 ± 4.33	29.53 ± 4.19
Subsets	R_1.2I_5.5;		
for α = 0.05	R_1.8I_6;		
	R_2.4I_7		
*V*_45Gy_ (%)	21.66 ± 5.73	22.05 ± 5.50	23.20 ± 4.07
Subsets	{R_2.4I_7, R_1.8I_6, R_1.2I_5.5}		
for α = 0.05			

If 2 groups of plans belong to the same subset, it indicates that there is no statistically significant difference at the significance level of α = 0.05.

## 4. Discussion

The management of radiotherapy-related toxicities remains a key challenge in optimizing cervical cancer treatment. Our dosimetric analysis demonstrates that optimization margins of 1.2 to 2.4 cm, combined with iterative constraint adjustments, achieve a significant reduction in the small intestine Dmean by 1.21 to 1.83 Gy (*P* < .001).

Our methodology introduces 2 key innovations: First, we established a margin-dependent constraint gradient model, revealing that larger margins require 30% to 40% stronger constraints to achieve comparable D_mean reductions (e.g., R_2.4I_7 requires 1.5 additional iterations vs R_1.2I_5.5). This phenomenon correlates with dose gradient characteristics – each 1 cm increase from PTV reduces 80% isodose line expansion by 2.1 ± 0.3 mm (Monte Carlo simulation data not shown).^[15]^ Second, the proposed dynamic iteration termination criteria (D_mean_ change < 0.3 Gy/2 iterations or PTV V_95%_ <98%) effectively balances intestinal sparing and target coverage, reducing optimization time by 17% compared to fixed iteration methods.^[16]^

DVH analysis demonstrates that margin selection significantly influences dose distribution patterns: smaller margins (1.2cm) predominantly reduce intermediate-high-dose regions (V_30Gy_ decreased by 2.7%, V_40Gy_ decreased by 1.1%), while larger margins (2.4cm) more effectively control low-dose exposure (V_15Gy_ decreased by 3.3%). There is a notable difference in research findings regarding intestinal dose-volume limitations and Grade 2 and 3 acute adverse reactions. For rectal cancer, a low-dose volume of the small intestine has been identified as a significant predictor of intestinal adverse reactions, with key indicators such as V_15Gy_.^[4]^ Conversely, studies on prostate cancer and certain cervical cancers have demonstrated that high-dose volumes are associated with the occurrence of intestinal adverse reactions.^[5,6]^ This difference may be attributed to the larger volume of rectal radiation in rectal cancer patients, whereas treatments for cervical and prostate cancers aim to minimize exposure to the rectum. Additionally, rectal cancer radiotherapy typically involves less localized dose escalation in the target area, resulting in smaller high-dose volume irradiation. On the other hand, cervical and prostate cancer treatments often include higher high-dose volumes in the intestine due to localized dose escalation in lymph nodes and primary lesions.

Therefore, clinical decision-making should be tailored to individual patient characteristics. For patients with lymph node metastasis (requiring localized dose escalation in lymph nodes), prioritizing a 1.2 cm margin to control V_40Gy_ is advisable. For patients with poor performance status (lower tolerance to acute toxicity), a 2.4 cm margin may be more appropriate.

Three main limitations warrant consideration: Single bowel delineation method using bowel bag contouring, though demonstrating superior reproducibility (ICC = 0.92 vs 0.76 for bowel loop method),^[17]^ may overestimate doses by 5% to 8% (unpublished data). Future studies should integrate DWI-MRI for high-risk segment identification.^[18]^ The positioning constraints also influence the outcomes. Supine positioning with a filled bladder improves setup reproducibility (with changes in PTV coverage of <2%) but may increase anterior small bowel exposure by 10% to 15%.^[19]^ Additionally, the absence of biological modeling presents a limitation. While physical dose parameters improved significantly, NTCP modeling (e.g., Lyman-Kutcher predictions showing ≥ 2 grade enteritis risk reduction from 28% to 19%)^[20]^ remains essential for clinical benefit quantification.

Future directions should focus on developing adaptive margin algorithms incorporating anatomical variables (BMI, uterine anteversion); implementing multimodal image-guidance with daily CBCT for bowel tracking; and exploring proton therapy advantages, with preliminary data showing additional D_mean_ reduction of 2.3 to 3.1 Gy (*P* = .002).^[21]^

## 5. Conclusion

This study establishes an effective framework for intestinal dose optimization in cervical cancer radiotherapy through strategic optimization structure implementation. The identified optimal margin range (1.2–2.4 cm) effectively achieves a clinically meaningful reduction in small intestine dose (mean dose difference: 1.5 ± 0.3 Gy) while preserving target coverage integrity.

Supplemental Digital Content “Study’s minimal underlying data set” is available for this article (https://links.lww.com/MD/Q106).

## Acknowledgments

The authors would like to express their gratitude to Peng Zhang from the Department of Radiology at Tianjin Haihe Hospital, Tianjin, China, for providing technical guidance.

## Author contributions

**Conceptualization:** Rui Ban, Jing Zeng, Jinlong Hao.

**Data curation:** Rui Ban, Jing Zeng, Jinlong Hao.

**Formal analysis:** Rui Ban, Jing Zeng, Jinlong Hao.

**Funding acquisition:** Rui Ban, Jing Zeng, Jinlong Hao.

**Investigation:** Rui Ban, Jing Zeng, Jinlong Hao.

**Methodology:** Rui Ban, Jing Zeng, Jinlong Hao.

**Project administration:** Rui Ban, Jing Zeng, Jinlong Hao.

**Resources:** Rui Ban, Jing Zeng, Jinlong Hao.

**Software:** Rui Ban, Jing Zeng, Jinlong Hao.

**Supervision:** Rui Ban, Jing Zeng, Jinlong Hao.

**Validation:** Rui Ban, Jing Zeng, Jinlong Hao.

**Visualization:** Rui Ban, Jing Zeng, Jinlong Hao.

**Writing – original draft:** Rui Ban, Jing Zeng, Jinlong Hao.

**Writing – review & editing:** Rui Ban, Jing Zeng, Jinlong Hao.

## Supplementary Material
